# Supplementary material for: Paramagnetic Properties and Moderately RapidConformational Dynamics in the Cobalt(II) Calix[4]arene Complex by NMR
Source: Molecules. 2022 Jul 21;27(14):4668. doi: 10.3390/molecules27144668 (PMC9317762; doi:10.3390/molecules27144668)
Supplement: Supplementary file 1 [file molecules-27-04668-s001.zip › molecules-1809804-supplementary.pdf]

Supplementary material

to the paper

Paramagnetic Properties and Moderately Rapid Conformational Dynamics in Cobalt(II) Calix[4]arene Complex by NMR

Eugeny N. Zapolotsky[a*], Sergey P. Babailov[a], Gennadiy A. Kostin[a],

[a] *A.V.Nikolayev's Institute of Inorganic Chemistry SB RAS, Ave. Lavrentyev, 3, Novosibirsk, 630090, Russia*

* Author for correspondence zapolotsky.en@gmail.com

Determination of rate constants

The rate constants of chemical exchange were found using approximate formulas for the cases of slow (1) and fast two-position chemical exchange (2):

$$k_A = \pi W_A - \frac{1}{T_{2A}}, \quad (1)$$

$$k_A = \frac{\pi}{2}\frac{\delta\nu^2}{(W_A - \frac{1}{T_{2A}})} \quad (2)$$

where k_A is the chemical exchange rate constant, W_A is the half-width of the signal peak at half-height, T_{2A} is the signal transverse relaxation time, δν is the difference between the resonant frequencies of the exchanged signals, expressed in Hz. It was assumed that the value of the transverse relaxation time T_{2A} of the corresponding diamagnetic state does not depend significantly on the temperature.

Calculation of lanthanide induced shifts (LIS) depending on the temperature $\delta_i(T)$ for various positions in the absence of exchange is carried out according to the formula:

$$\delta_i(T) = \delta_i(T_0)\frac{(1/T + A)}{(1/T_0 + A)}, \qquad (3)$$

where $\delta_i(T_0)$ is the LIS value corresponding to a given position at a low temperature T_0 = 273.2K; A is a constant determined from the analysis of the temperature dependence of the average value of LIS for all protons involved in the exchange.

The value of parameter A was determined by the formula (4):

$$A = \frac{\delta_i(T_0)/T - \delta_i(T)/T_0}{\delta_i(T) - \delta_i(T_0)} \qquad (4)$$

The values of δν at different temperatures were estimated by taking into account the temperature dependence of LIS for each of the signals of paramagnetic Ln complexes involved in chemical exchange.

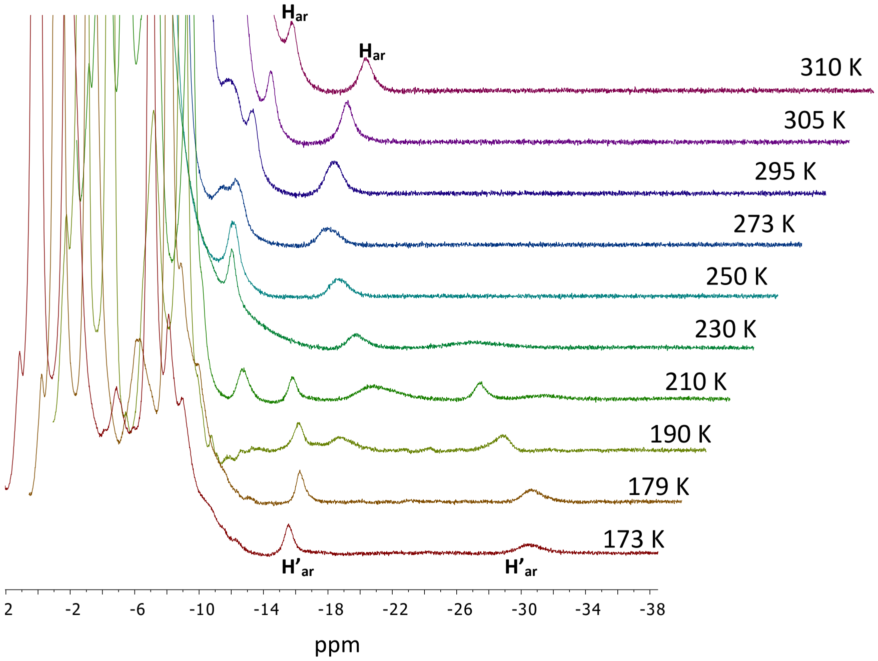

Figure S1. ^{1}H NMR spectra of Co calix[4]arene at different temperature.

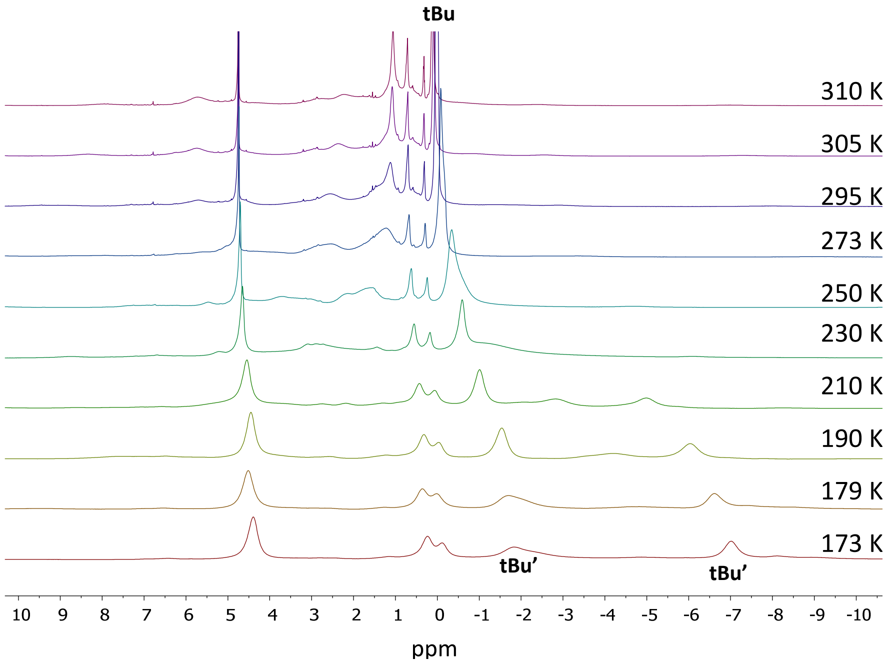

Figure S2. ^{1}H NMR spectra of Co calix[4]arene at different temperature.

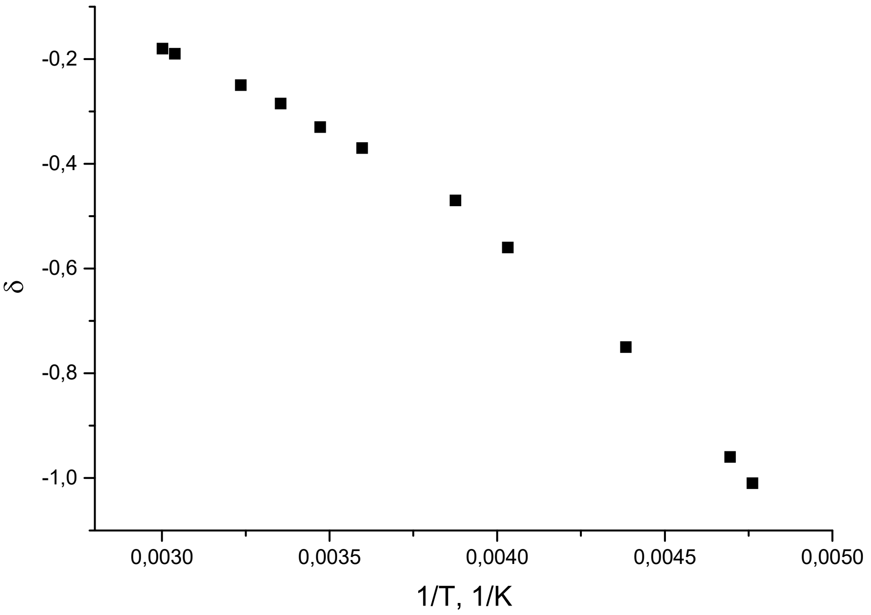

Figure S3. Temperature dependence of the chemical shifts of t-Bu signal of complex (**I**). The solvent is $CDCl_3$/ CD_2Cl_2.
